# Case Report: Neurodegenerative Diseases After Severe Acute Respiratory Syndrome Coronavirus 2 Infection, a Report of Three Cases: Creutzfeldt–Jakob Disease, Rapidly Progressive Alzheimer's Disease, and Frontotemporal Dementia

**DOI:** 10.3389/fneur.2022.731369

**Published:** 2022-02-07

**Authors:** Gabriela Almeida Pimentel, Thiago Gonçalves Guimarães, Guilherme Diogo Silva, Milberto Scaff

**Affiliations:** ^1^Hospital Sirio-Libanes and The University of São Paulo Medical School, Neurology, São Paulo, Brazil; ^2^Hospital Sirio-Libanes and Professor of The University of São Paulo Medical School, Neurology, São Paulo, Brazil

**Keywords:** SARS-CoV-2, COVID-19, neurodegenerative disease, case report, Creutzfeldt–Jakob disease, Alzheimer's disease, frontotemporal dementia

## Abstract

The relationship between severe acute respiratory syndrome coronavirus 2 (SARS-CoV-2) and neurodegenerative diseases is yet to be fully clarified. Rapid worsening and even new-onset cases of those disorders have been reported in association with coronavirus disease 2019 (COVID-19). We describe three cases of neurodegenerative diseases in patients with SARS-CoV-2: a case of Creutzfeldt–Jakob disease during the COVID-19 acute phase, to our knowledge, is the second one described in the literature; a rapidly progressive Alzheimer's Disease; and a patient with frontotemporal dementia, and a quick decline of both cognitive and behavioral domains. This report suggests an association between SARS-CoV-2 infection and a higher probability of developing or accelerating neurodegenerative chronic neurologic conditions. We reinforce the need for a close cognitive follow-up in the aftermath of Sars-Cov2 infection.

## Introduction

Patients with coronavirus disease 2019 (COVID-19) present neurological manifestations such as dizziness, headache, and impaired consciousness (1). The impact on cognitive symptoms is still under debate.

Even mild forms of COVID-19 can present sustained neurocognitive deficits (2, 3). Furthermore, patients previously diagnosed with dementia may be at risk of rapid cognitive deterioration during and after severe acute respiratory syndrome coronavirus 2 (SARS-CoV-2) infection (4).

Mild forms of frontotemporal dementia (FTD) may have significant worsening in behavior and social cognition after COVID-19 (5). Recently, Creutzfeldt–Jakob disease (CJD) was reported in a previously healthy man during the acute phase of COVID-19 (6).

We report three cases of neurodegenerative cognitive diseases in patients with COVID-19, and discuss the relationship between CJD, Alzheimer's Disease (AD), FTD, and SARS-CoV-2 infection.

## Case Presentation 1

A 75-year-old man presented with a 15-day history of confusion, agitation, suicidal ideation, and inability to walk. He did not have any history of previous behavioral or cognitive symptoms. A brain magnetic resonance imaging (MRI) and lumbar puncture were performed. No abnormalities were identified at that time.

On the following days, he developed fever, and an RT-PCR for SARS-CoV-2 tested positive. At that time, chest CT revealed ground-glass opacities of more than 50% of lung territory. A further deterioration of respiratory function led to orotracheal intubation. He was treated with methylprednisolone, sulfamethoxazole-trimethoprim, meropenem, and linezolid for associated bacterial pneumonia. Acute renal failure was managed with hemodialysis.

He was transferred to our service for further investigations and treatment. Neurologic examination revealed impaired consciousness, bilateral spastic hemiparesis, and myoclonic jerks. No other abnormalities were detected.

**Figure 1:**
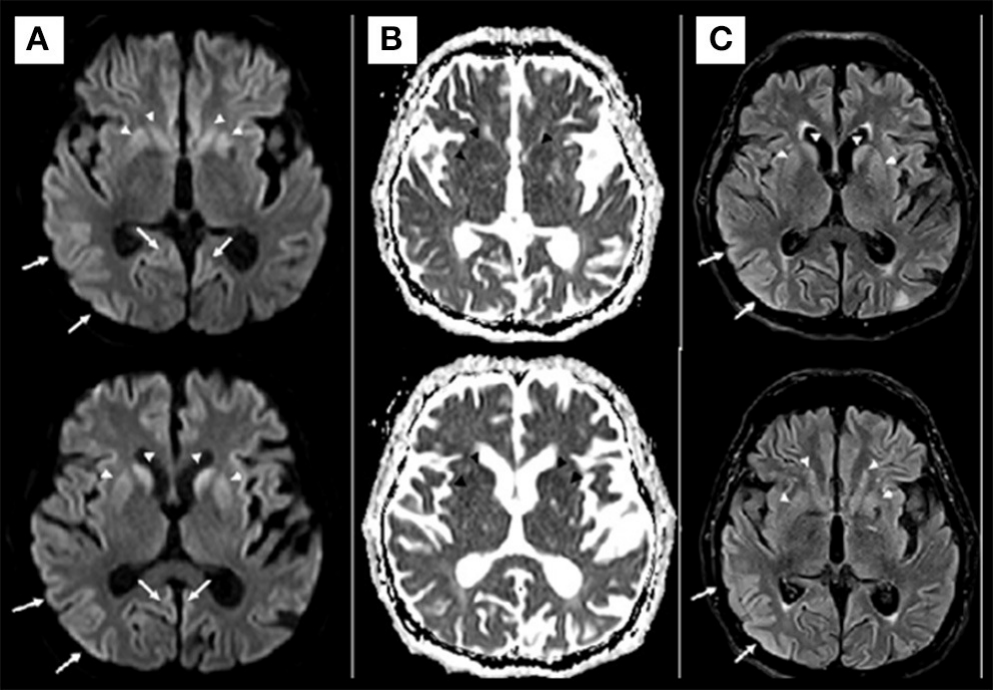
Axial diffusion [diffusion weighted imaging (DWI)] **(A)** and apparent diffusion coefficient (ADC) map **(B)** MRI at admission. Diffusion restriction involving the cerebral cortex and basal ganglia (arrows in **A**). No convincing white matter involvement. The hypotheses for MRI findings were pos-ictal state, hypoxic-ischemic encephalopathy, and spongiform encephalopathy. Axial Diffusion and FLAIR **(C)** MRI after 2 weeks demonstrated similar imaging findings, making the hypothesis of spongiform encephalopathy more likely.

**Figure 2:**
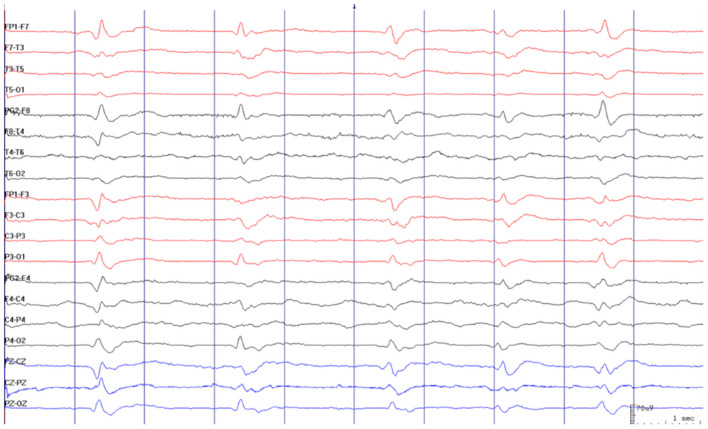
Electroencephalogram revealed 1–2 Hz generalized periodic discharges (GPDs) and diffuse theta-delta slowing.

Brain MRI showed restricted diffusion with corresponding FLAIR hyperintensity diffusely in the cortex and striate nucleus, sparing pre-central gyrus and hippocampus (Figure 1). Cerebrospinal fluid (CSF) had a normal cell count, and a mildly elevated protein level. Glucose, bacterial culture, molecular testing for herpes virus, and SARS-CoV-2 PCR performed in the CSF were negative. Electroencephalogram (EEG) revealed 1-2 Hz generalized periodic discharges (GPDs) and a diffuse theta-delta slowing (Figure 2).

The patient was treated with intravenous immune globulin (IVIG) until CSF, and the serum autoimmune encephalitis panels resulted negative. After 3 weeks, positive 14-3-3, T-TAU (> 20,000 pg/ml), and CSF RT-QuIC tests reinforced the hypothesis of probable sporadic CJD (7, 8). Unfortunately, he died of sepsis, secondary to bacterial pneumonia 4 months after the symptoms onset.

There was no family history of CJD or any other dementia. No risk factors for iatrogenic CJD such as previous neurosurgery and blood transfusions were reported.

## Case Presentation 2

A 69-year-old female, with neither cognitive nor psychiatric antecedents, started having symptoms of panic disorder and complaints of forgetfulness (name of family members and friends, appointments, addresses) 1 month after a mild COVID-19 infection. There was no family history of neurological conditions.

Her past medical history was remarkable only for hypercholesterolemia and hypothyroidism. The patient had no history of smoking, abusive alcohol intake, or illicit drug use. A rapidly progressive dementia diagnostic workup excluded infectious, metabolic, and inflammatory causes.

Neurocognitive assessment at the first clinic visit revealed a Montreal Cognitive Assessment test of 7/30. Her speech was non-fluent with word-finding difficulties. Semantic and phonemic verbal fluency was 6 and 5 words per minute, respectively. Additionally, the patient had trouble in both naming objects and repeating sentences. Memory was also impaired on bedside testing, the Figure Memory Test (9). The rest of the neurological examination was unremarkable.

**Figure 3:**
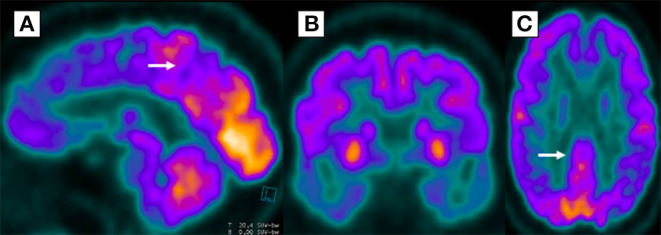
Diffuse glycolytic hypometabolism in posterior parietal regions, extending bilaterally to precuneus **(A)**, temporal **(B)**, posterior cingulate gyrus **(C)**, and frontal lobes in 18-fluorodeoxyglucose positron emission tomography.

Brain MRI did not show any structural abnormality that could explain the cognitive decline. The FDG-PET showed diffuse cerebral hypometabolism in the posterior parietal regions with extension to the posterior cingulate gyrus and bilateral pre-cuneus, temporal, and frontal lobes, suggesting Alzheimer's disease (Figure 3). Amyloid biomarkers such as Pittsburgh compound-B positron emission tomography scan (PiB-PET) and CSF Aβ42 levels were not available at our service; therefore, they could not be performed. However, according to the National Institute on Aging and the Alzheimer's Association (NIA-AA), the patient still fulfilled the criteria for possible AD dementia (10).

She was started on acetylcholinesterase inhibitors and remained stable at 1-year follow-up.

## Case Presentation 3

A 55-year-old man was hospitalized due to a clinical syndrome suggestive of COVID-19 (cough, anosmia, and fever). After discharge, he developed progressive difficulty in paying bills, carrying out domestic activities, and organizing activities at work in the last 5 months.

Family members informed that the patient had been showing behavioral changes, episodes of apathy, excessive money spending, and social inadequacy for the past 2 years, which led to the end of a romantic relationship. These behavioral changes were treated as depressive symptoms with sertraline 50 mg/day. Subsequently, clonazepam 1 mg/day was started for an associated anxiety disorder, with no significant response. There was no other remarkable personal or family medical history.

Upon cognitive examination, the patient scored 17/30 on the Mini-Mental State Examination, which revealed impairment in executive function and language. In the clock drawing test, the patient wrote numbers from 1 to 24. The rest of the neurological examination was unremarkable.

**Figure 4:**
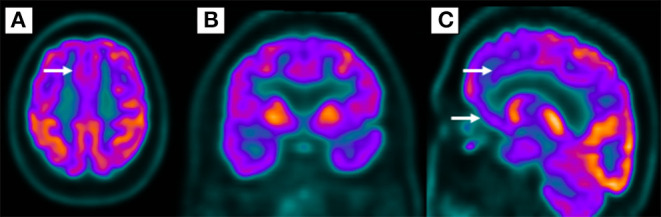
Bilateral frontal hypometabolism **(A,B)**, more evident in the medial and mid-basal frontal lobe **(C)** in 18-fluorodeoxyglucose positron emission tomography.

The patient underwent a brain MRI, which was unremarkable. Then, a cerebral PET-FDG was performed, which revealed a moderate bilateral frontal hypometabolism, more evident in medial and mid-basal appearance, including anterior cingulate gyrus, and, to a lesser extent, temporal lobes, supporting the hypothesis of FTD (Figure 4).

The patient fulfilled the criteria for a probable behavioral variant of FTD: executive dysfunction, apathy, impulsivity (impulsive purchases), loss of empathy (social inadequacy with the end of a loving relationship), and a suggestive PET-FDG. Unfortunately, after hospital discharge, the patient was lost to follow-up.

## Discussion

The SARS-CoV-2 is detected in the central nervous system (CNS) in patients with COVID-19 (11). Coronavirus accesses the CNS *via* the olfactory bulb and cerebral vasculature through the angiotensin-converting enzyme 2 (ACE-2) receptor (12).

The CNS damage is probably attributable to systemic inflammation, peripheral organ dysfunction, and cerebrovascular changes. Hyperinflammation with elevated interleukin-1β, interferon-γ (INF-γ), C-reactive protein (CRP), granulocyte colony-stimulating factor (G-CSF), CXCL10, monocyte protein 1-α, and tumor necrosis factor-α (TNF-α) was described during COVID-19 infection (13, 14).

Recently, a case-control study has found that elevated CRP levels were associated with cognitive dysfunction in patients with COVID-19, specifically sustained attention. Authors cited previous works that suggested CRP might have an early effect on frontal lobe functioning and highlighted the association of other viral infections with cognitive impairment (15).

Studies on sepsis have associated systemic inflammation with cognitive decline and neurodegenerative disease (16–19). A condition related to severe systemic inflammation, COVID-19, may similarly increase the risk to develop or accelerate the subclinical neurodegenerative conditions.

Both SARS-CoV-2 and AD present a similar inflammatory markers profile: IL-6, IL-1, GAL-9 (galectin-9), and CKAP4 (cytoskeleton-associated protein 4). ACE-2 receptor, and SARS-CoV-2-binding protein for cell entry, was found to have a ten-time higher expression in AD brains than controls (20). The presence of APOE-ϵ4 mutation, the strongest genetic risk factor for AD, was associated with an increased risk of infection and mortality due to COVID-19 (21).

The FTD also presents a pro-inflammatory cytokine signature with increased IL-6 and IL-1 (22), similar to COVID-19. The progranulin levels, a protein mutated in familial forms of FTD, were associated with the severity of COVID-19 (23).

The SARS-CoV-2 contributes to chronic neurological damage through hypoxia and cerebral hypoperfusion secondary to: (a) cardiorespiratory disease, (b) coagulopathy resulting in thrombotic occlusion of cerebral blood vessels, (c) and cerebral microvascular damage due to endothelial dysfunction. Cerebral hypoperfusion accelerates amyloid-β (Aβ) accumulation and is linked to tau and TDP-43 pathology (12).

This paper has several limitations. None of the cases had anatomopathological confirmation, amyloid biomarkers were unavailable for Patient 2, and Patient 3 was lost to follow-up. However, we believe that sufficient data on clinical history and complementary exams could be gathered to exclude differentials and fulfill either possible or probable diagnosis based on published criteria (7, 8, 10, 24).

Although far from establishing a causal relationship, our report may be added to the previous and future ones in the hope of building more robust evidence of association between SARS-CoV-2 and a higher probability of developing or accelerating the neurodegenerative chronic neurologic conditions. We believe this association may be explained by inflammatory and vascular mechanisms. Furthermore, it reinforces the need for cognitive follow-up after the infection is resolved, especially in older patients at risk of developing dementia.

## Data Availability Statement

The original contributions presented in the study are included in the article/Supplementary Material, further inquiries can be directed to the corresponding author.

## Ethics Statement

Written informed consent was obtained from the individual(s) for the publication of any potentially identifiable images or data included in this article.

## Author Contributions

MS revised the case report for intellectual content. All authors contributed to the article and approved the submitted version.

## Conflict of Interest

The authors declare that the research was conducted in the absence of any commercial or financial relationships that could be construed as a potential conflict of interest.

## Publisher's Note

All claims expressed in this article are solely those of the authors and do not necessarily represent those of their affiliated organizations, or those of the publisher, the editors and the reviewers. Any product that may be evaluated in this article, or claim that may be made by its manufacturer, is not guaranteed or endorsed by the publisher.
