# Supplementary material for: Case Report: Neurodegenerative Diseases After Severe Acute Respiratory Syndrome Coronavirus 2 Infection, a Report of Three Cases: Creutzfeldt–Jakob Disease, Rapidly Progressive Alzheimer's Disease, and Frontotemporal Dementia
Source: Front Neurol. 2022 Feb 7;13:731369. doi: 10.3389/fneur.2022.731369 (PMC8858976; doi:10.3389/fneur.2022.731369)
Supplement: Supplementary file 1 [file Data_Sheet_1.PDF]

CARE Checklist of information to include when writing a case report

CARE case report guidelines

(cc) BY-NC-ND

Topic	Item	Checklist item description	Reported on Line
Title	1	The diagnosis or intervention of primary focus followed by the words "case report"	P3
Key Words	2	2 to 5 key words that identify diagnoses or interventions in this case report, including "case report"	P3
Abstract (no references)	3a	Introduction: What is unique about this case and what does it add to the scientific literature?	P3
	3b	Main symptoms and/or important clinical findings	P3
	3c	The main diagnoses, therapeutic interventions, and outcomes	P3
	3d	Conclusion—What is the main "take-away" lesson(s) from this case?	P3
Introduction	4	One or two paragraphs summarizing why this case is unique (**may include references**)	P4
Patient Information	5a	De-identified patient specific information	P4; P5; P6
	5b	Primary concerns and symptoms of the patient	P4; P5; P6
	5c	Medical, family, and psycho-social history including relevant genetic information	P4; P5; P6
	5d	Relevant past interventions with outcomes	P4-5; P5; P6
Clinical Findings	6	Describe significant physical examination (PE) and important clinical findings	P5; P6; P6
Timeline	7	Historical and current information from this episode of care organized as a timeline	NA*
Diagnostic Assessment	8a	Diagnostic testing (such as PE, laboratory testing, imaging, surveys).	P5; P6; P7
	8b	Diagnostic challenges (such as access to testing, financial, or cultural)	P5; P6; P7
	8c	Diagnosis (including other diagnoses considered)	P5; P6; P7
	8d	Prognosis (such as staging in oncology) where applicable	P5; P6; P7
Therapeutic Intervention	9a	Types of therapeutic intervention (such as pharmacologic, surgical, preventive, self-care)	P5; P6; NA#
	9b	Administration of therapeutic intervention (such as dosage, strength, duration)	P5; P6; NA#
	9c	Changes in therapeutic intervention (with rationale)	P5; NA; NA#
Follow-up and Outcomes	10a	Clinician and patient-assessed outcomes (if available)	NA#
	10b	Important follow-up diagnostic and other test results	P5; P6;NA#
	10c	Intervention adherence and tolerability (How was this assessed?)	NA#; P6; NA#
	10d	Adverse and unanticipated events	P5; P6; NA#
Discussion	11a	A scientific discussion of the strengths AND limitations associated with this case report	P8
	11b	Discussion of the relevant medical literature **with references**	P7-8
	11c	The scientific rationale for any conclusions (including assessment of possible causes)	P7-8
	11d	The primary "take-away" lessons of this case report (without references) in a one paragraph conclusion	P8
Patient Perspective	12	The patient should share their perspective in one to two paragraphs on the treatment(s) they received	NA#
Informed Consent	13	Did the patient give informed consent? Please provide if requested	Yes ☐ No [x]

Observations:

* 7 : We believe timelines are particularly useful in single case reports. For case series, including 3 patients, we did not find this would improve readability.

# 9-10d, 12: data is not available due to loss of follow up in patient three or premature death in patient 1.

We present only de-identified patient data.
